# Bronchial varices in a child with tricuspid atresia six years post Fontan correction

**DOI:** 10.1002/rcr2.620

**Published:** 2020-07-16

**Authors:** Shreya Bhushan, Vikas Goyal, Cameron Ward, Muddassir Rashid, Nitin Kapur

**Affiliations:** ^1^ Department of Respiratory and Sleep Medicine Queensland Children's Hospital Brisbane QLD Australia; ^2^ Department of Cardiology Queensland Children's Hospital Brisbane QLD Australia; ^3^ Department of Interventional Radiology Queensland Children's Hospital Brisbane QLD Australia

**Keywords:** Bronchial varices, bronchoscopy, embolization, Fontan

## Abstract

Tracheal and bronchial varices are rarely found in children. However, they have been described in adults with failing Fontan circuits or secondary to vascular pathology, such as portal and pulmonary hypertension. We report the presentation of haemoptysis and bronchial varices in a child, six years after a Fontan procedure for tricuspid atresia. She had tortuous mediastinal and transpleural arterial collaterals on computed tomography (CT) angiography and cardiac catheterization and subsequently underwent embolization of these collaterals. While the haemoptysis settled post embolization, the bronchial varices persisted on repeat bronchoscopy. She has since been clinically well with no further haemoptysis.

## Graphical abstract

Tracheal and bronchial varices are rare, especially in children. We report the first presentation of bronchial varices and haemoptysis in a child, six years after a Fontan procedure for tricuspid atresia. She had tortuous mediastinal and transpleural arterial collaterals on imaging and subsequently underwent embolization of these collaterals with haemoptysis settling after this.

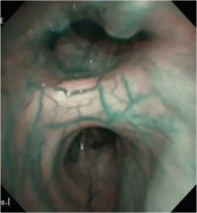

## Introduction

The Fontan procedure redirects blood from the inferior and superior vena cavae to the pulmonary arteries, bypassing the heart [1]. It is used as palliative surgery for children with a univentricular heart, including tricuspid atresia. However, it has a known high delayed failure rate with development of atrial arrhythmia, protein‐losing enteropathy, plastic bronchitis, cardiac failure, exercise intolerance, reduced life expectancy, cirrhotic liver disease, hepatic carcinoma, schooling difficulties, progressive cyanosis, and systemic venous hypertension [2, 3]. The case presented here is the first reported case of bronchial varices in a child with tricuspid atresia post Fontan correction.

## Case Report

A 12‐year‐old female patient with palliated congenital heart disease presented to emergency with an episode of atraumatic haemoptysis that resolved spontaneously. She had a history of tricuspid atresia type 1C with a large ventricular and atrial septal defect. She initially underwent pulmonary artery banding at six weeks of age, with a subsequent bidirectional Glenn (rerouting of the superior vena cava to the right pulmonary artery) and closure of her atrial septal defect (ASD) and ventricular septal defect (VSD). The decision was made in theatre by the surgical team to insert a homograft valve between the right atrium and right ventricle. However, this was complicated by severe homograft regurgitation with resultant protein‐losing enteropathy. She subsequently underwent removal of the homograft valve, perforation of her VSD patch, and creation of an ASD. Her protein‐losing enteropathy significantly improved over time and she went on to have a successful Fontan procedure with extracardiac conduit and inclusion of a 4‐mm fenestration at eight years of age.

Four years after the Fontan procedure, the patient presented to emergency with an episode of spontaneous atraumatic small‐volume haemoptysis, not brought on by exertion. This was not associated with dyspnoea, chest pain, or significant cough. There were no infective symptoms or melaena. She had no obvious respiratory distress and a clear chest on auscultation. Her vital signs were normal besides a resting saturation of 91% (her baseline). On auscultation, she had a single second heart sound, although with no murmurs. Autoimmune blood examinations done for vasculitis were normal. Her haemoglobin was 152 g/L with a platelet count of 282 × 10^9^/L and normal coagulation studies with mild transaminitis.

**Figure 1:**
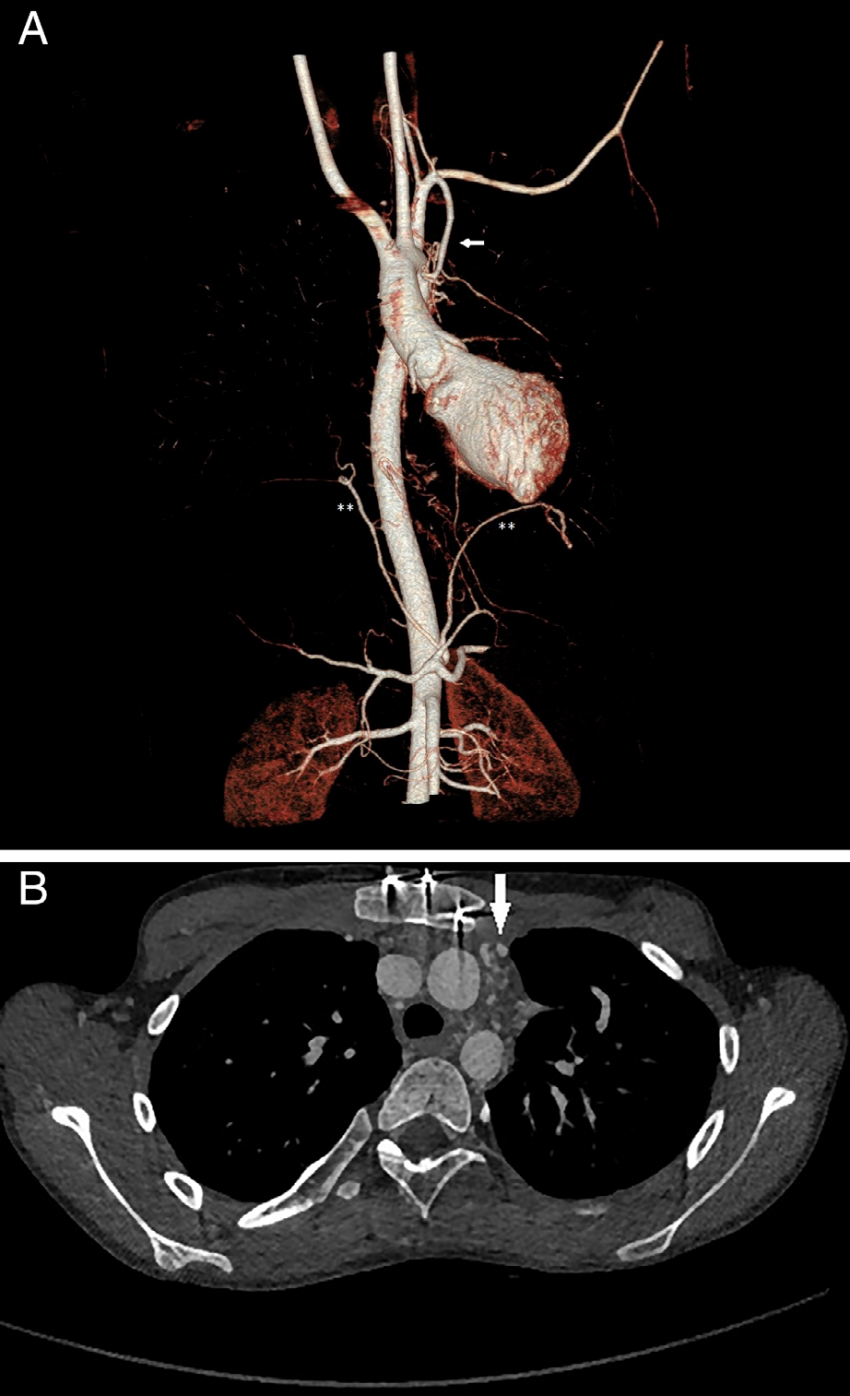
Computed tomography (CT) angiogram of the chest with (A) volume rendering technique and (B) axial images demonstrating hypertrophied left internal mammary (arrow) and bilateral inferior phrenic arteries (**) supplying collaterals to bronchi in the superior mediastinum. No ground‐glass changes were noted to suggest pulmonary haemorrhage.

Cardiac computed tomography (CT) showed widely patent Fontan and Glenn shunts but tortuous mediastinal and trans‐pleural collaterals, with largest feeding vessels extending from the right subclavian (2.3 mm) and phrenic arteries (1.6 mm) and left internal thoracic (1.7 mm) and phrenic arteries (2.2 mm) to the pulmonary vascular bed (Fig. 1). Lung windows indicated multiple small collateral vessels with prominent interstitium throughout the lung fields, right more than left. There was no endoluminal debris or ground‐glass change noted on CT or chest X‐ray.

**Figure 2:**
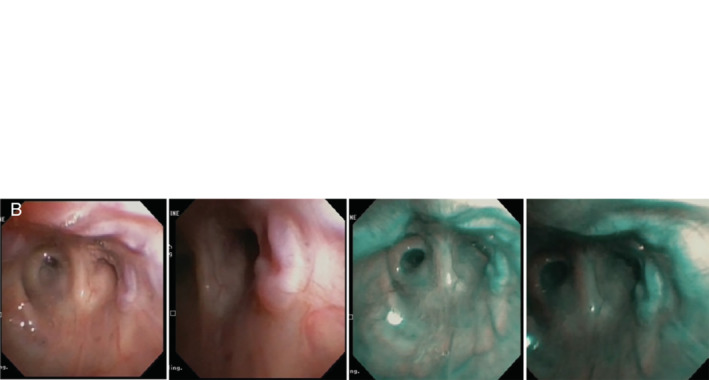
Bronchoscopy findings with (A) engorged vessels in the right upper lobe and (B) persistence of bronchial varices after embolization of hypertrophic collaterals.

Flexible bronchoscopy showed prominent bronchial varices in the right upper lobe (RUL) bronchi with tortuous vessels in the right main stem bronchus, bronchus intermedius, and right middle lobe bronchus. She had especially engorged and tortuous submucosal vessels on the roof and floor of the RUL extending into the anterior and posterior segmental bronchi of the RUL bronchi (Fig. 2).

The bronchoalveolar lavage fluid (BALF) from the lingula cultured *Pseudomonas aeruginosa*, which was treated with dual anti‐pseudomonal therapy. BALF was not stained to detect haemosiderin‐laden macrophages (HLM). A gastroscopy done at the same time did not show any oesophageal or gastric varices.

As her coagulation and connective tissue screens were negative, it was deemed unlikely that the child had diffuse alveolar haemorrhage or a pulmonary vasculitic process. Echocardiogram suggested the possibility of mildly increased Fontan pressures with mean gradient through the fenestration being 8 mmHg. However, cardiac catheterization demonstrated mean Fontan pressures of only 7 mmHg with a transpulmonary gradient of 4 mmHg and femoral arterial saturations of 91% with no major aortopulmonary or innominate artery collaterals. The branch pulmonary arteries were unobstructed on both CT and angiography.

She had two further episodes of self‐resolving small‐volume fresh haemoptysis in the 12 months after the first episode, with the second being associated with a three‐day history of a productive green cough and shortness of breath, which was treated with intravenous anti‐Pseudomonal antibiotics.

**Figure 3:**
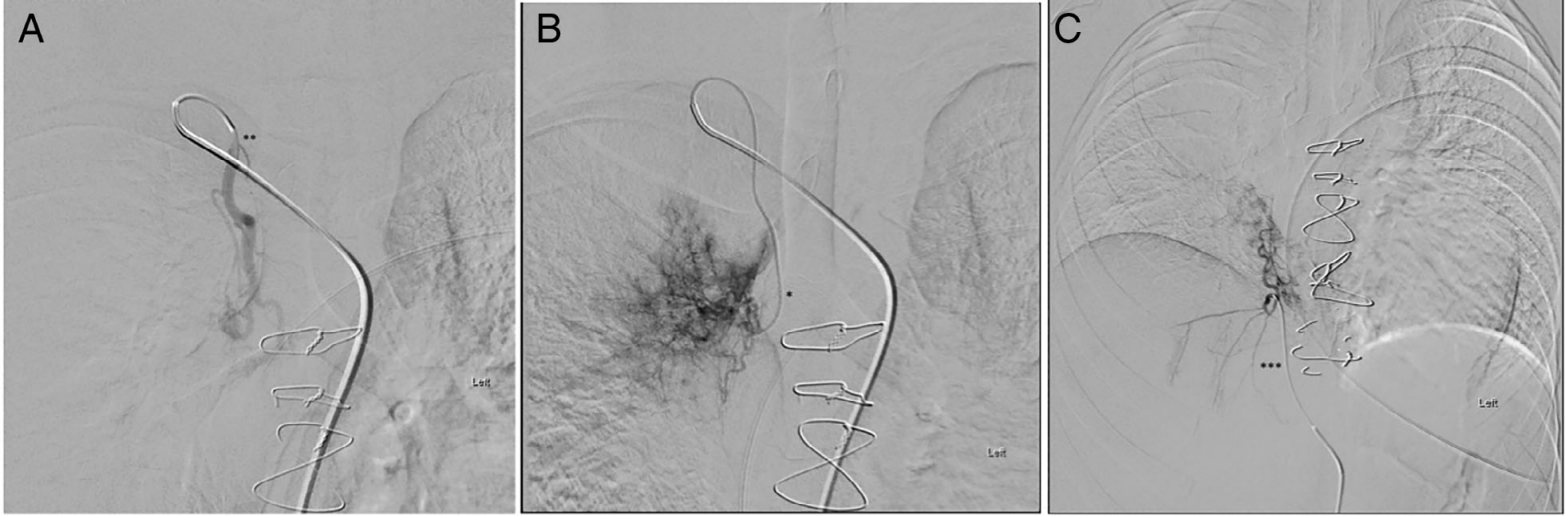
Catheter angiograms showing (A) the hypertrophic right internal mammary artery (**) with (B) a microcatheter placed deep in the vessel opacifying abnormal systemic collaterals (*) and (C) selective angiogram of the right inferior phrenic artery (***) demonstrating systemic collaterals in the right lung.

She had a repeat bronchoscopy, which showed persistent bronchial varices in the RUL. The second BALF was obtained from the right middle lobe and lingula and showed HLMs on Perl's Prussian blue staining. CT angiography showed persistent hypertrophic collaterals from the right costocervical branch, right phrenic, and left internal mammary artery. Hypertrophic systemic arterial collaterals were also seen along proximal airways on digital subtraction angiography. These vessels were selectively catheterized and abnormal collaterals embolized with polyvinyl alcohol (PVA) particles by interventional radiology (Fig. 3). Bronchial varices persisted on bronchoscopy despite the embolization, although the haemoptysis did not recur. Months onwards, she has been stable from a respiratory and cardiac perspective. A spirometry done six months post‐embolization showed a forced expiratory volume in 1 sec (FEV_1_) of 1.72 L (59% predicted), with a forced vital capacity (FVC) of 2.09 L (64% predicted) and FEV_1_/FVC of 83%, suggesting a restrictive defect. This was attributed to her underlying scoliosis.

## Discussion

Bronchial varices are abnormally dilated, tortuous blood vessels in the pulmonary system and are a rare cause of haemoptysis in children. They can occur due to prolonged increased pulmonary vascular resistance, including portal and pulmonary hypertension [4]. Pulmonary or portal vein stenosis [5, 6], pulmonary vein atresia [7], chronic liver congestion [8], cirrhosis [9, 10], and portal venous thrombosis [11] have all been reported to cause tracheal‐bronchial varices. Many children with cyanotic heart disease develop collateral circulations as a physiological response to relative hypoxia or altered pulmonary flow, with arterial collaterals commonly seen pre‐ and post‐Fontan [12].

Aortopulmonary collaterals have been found on angiography in 37% of catheterizations in patients post a Fontan or bidirectional Glenn surgery [13]. Although development of subsequent haemoptysis is uncommon, it has been reported in adults with major aortopulmonary collaterals and submucosal bronchial varices [2, 6, 14]. Venous collaterals, on the other hand, are commonly seen post bidirectional Glenn and Fontan due to the pressure gradient created between the higher pressure superior caval system and lower pressure veins supplying the receiving atrium [1, 15]. Development of haemoptysis because of these collaterals is also uncommon [4].

The Fontan procedure has greatly increased the life expectancy of patients with univentricular heart disease [2]. However, it has a known high delayed failure rate with a multitude of complications including atrial arrhythmia, protein‐losing enteropathy, cardiac failure, progressive cyanosis, exercise intolerance, systemic venous hypertension, cirrhotic liver disease, and hepatic carcinomas [3]. Recent case reports have also documented complications including the development of thromboembolic episodes, plastic bronchitis, and haemoptysis [2, 14]. However, bronchial varices post Fontan procedure are extremely rare and could be a sign of failing Fontan—a summary of available case reports is outlined in Table 1.

**Table 1:** Summary of case reports on tracheal or bronchial varices post Glenn or Fontan.

Study	Presentation	Postulated cause of varices	Intervention and outcomes
Machogu et al. (2013) [4]	A 14‐year‐old male with worsening haemoptysis due to tracheal varices on background of bidirectional Glenn for double‐inlet left ventricle	High pressures to SVC due to Glenn and anterograde pulmonary flow as well as heart failure resulting in cardiac cirrhosis and portal hypertension	Amplatzer vascular plugs with acute decompensation post catheterization due to haemoptysis. Required vasopressors and eventually heart and liver transplantation with complete resolution of varices post‐transplant
Sosa Lozano et al. (2011) [8]	A 32‐year‐old male with mild haemoptysis and SOB due to tracheal varices on background of Fontan for tricuspid atresia revised six years prior to presentation	Unclear	Conservatively managed for haemoptysis. The patient died from worsening left heart failure and multiorgan failure 14 months later
Patch et al. (2011) [16]	A 45‐year‐old male with bronchial varices and haemoptysis secondary to failing Fontan circulation 29 years post‐operatively	Liver venous outflow obstruction and early cirrhosis due to passive congestion	Conservative management of varices, awaiting transplantation
Bedard et al. (2008) [2]	A 19‐year‐old male with recurrent haemoptysis secondary to submucosal bronchial varices and aortopulmonary collaterals from bronchial arteries 10 years post Fontan	Unclear	Embolization of aortopulmonary collaterals arising from feeding bronchial arteries. Required extracorporeal membrane oxygenation as a bridge to left inferior lobectomy. Fontan modified with lateral tunnel approach with no recurrence of haemoptysis since then

SOB, Shortness of breath; SVC, Superior Vena Cava.

The precise aetiology of the bronchial varices and haemoptysis in our patient is unclear. Rather than a failing Fontan, our patient continues to have stable cardiac status with a robust Fontan circuit and low atrial, Fontan, inferior, and superior vena cava pressures three years after the varices were first identified. She had no signs of portal hypertension on imaging.

While conjectural, we hypothesize that the haemoptysis was possibly caused by the hypertrophic systemic arterial collaterals along her airways, rather than purely through bronchial varices. This is supported by the complete cessation of haemoptysis post embolization despite persistence of bronchial varices. It is also possible that the airway varices were receiving blood supply through a feeder systemic artery, although we did not find direct angiographic or CT evidence of the same. Shweihat and Zoby [17] reported similar resolution of haemoptysis in an adult with bronchial varices after embolization of feeding arteries, which also resulted in reduction of size of the varices. We also hypothesize that the varices relate to the patient's early surgery and homograft insertion, which failed, resulting in high right atrial pressures and protein‐losing enteropathy. High right atrial pressures would have caused portal hypertension with the liver having similar physiology as with a failing Fontan. Previous reports of bronchial varices in patients with portal hypertension have postulated decompression of the portal system through the tracheal varices [9]. As our patient had no evidence of oesophageal varices despite the period of significantly elevated portal venous pressure, this suggests an alternative mechanism for bronchial varices in the presence of elevated portal venous pressure, as seen in failing Fontan circuits. In our case, high portal pressures were relieved by the subsequent surgeries, resulting in good haemodynamics on repeat cardiac catherization. Despite the unclear origin of haemoptysis in our patient, this case emphasizes the importance of considering tracheal‐bronchial varices as a cause of haemoptysis in the setting of congenital heart disease associated with high portal venous pressures, such as in failing Fontan circuits.

### Disclosure Statement

Appropriate written informed consent was obtained for publication of this case report and accompanying images.
